# Low-Dimensional-Materials-Based Flexible Artificial Synapse: Materials, Devices, and Systems

**DOI:** 10.3390/nano13030373

**Published:** 2023-01-17

**Authors:** Qifeng Lu, Yinchao Zhao, Long Huang, Jiabao An, Yufan Zheng, Eng Hwa Yap

**Affiliations:** 1 School of CHIPS, XJTLU Entrepreneur College (Taicang), Xi’an Jiaotong-Liverpool University, 111 Taicang Avenue, Taicang, Suzhou 215488, China; 2 School of Intelligent Manufacturing Ecosystem, XJTLU Entrepreneur College (Taicang), Xi’an Jiaotong-Liverpool University, 111 Taicang Avenue, Taicang, Suzhou 215488, China; 3 School of Robotics, XJTLU Entrepreneur College (Taicang), Xi’an Jiaotong-Liverpool University, 111 Taicang Avenue, Taicang, Suzhou 215488, China

**Keywords:** artificial synapse, memristor, transistor, flexible electronics, neuromorphic computing, artificial perception system

## Abstract

With the rapid development of artificial intelligence and the Internet of Things, there is an explosion of available data for processing and analysis in any domain. However, signal processing efficiency is limited by the Von Neumann structure for the conventional computing system. Therefore, the design and construction of artificial synapse, which is the basic unit for the hardware-based neural network, by mimicking the structure and working mechanisms of biological synapses, have attracted a great amount of attention to overcome this limitation. In addition, a revolution in healthcare monitoring, neuro-prosthetics, and human–machine interfaces can be further realized with a flexible device integrating sensing, memory, and processing functions by emulating the bionic sensory and perceptual functions of neural systems. Until now, flexible artificial synapses and related neuromorphic systems, which are capable of responding to external environmental stimuli and processing signals efficiently, have been extensively studied from material-selection, structure-design, and system-integration perspectives. Moreover, low-dimensional materials, which show distinct electrical properties and excellent mechanical properties, have been extensively employed in the fabrication of flexible electronics. In this review, recent progress in flexible artificial synapses and neuromorphic systems based on low-dimensional materials is discussed. The potential and the challenges of the devices and systems in the application of neuromorphic computing and sensory systems are also explored.

## 1. Introduction

With the development of information technology and intelligent systems, the amount of data will double every two years, and these data need to be processed with high efficiency. However, the memory wall in conventional Von Neumann-based computing systems will limit their energy efficiency and their computing performance, due to the frequent transmission of data between memory and the processing unit [1,2]. In contrast, benefiting from large-scale parallel processing and event-driven operation of the biological neural network, the human brain is able to work in robust, fault tolerant, and energy-efficient modes [3,4,5], and only consumes about 20 W of power. In addition, some level activities, such as reflex and muscle activation, are processed directly by the peripheral neural system (PNS) without sending signals to the central neural system (CNS). That is to say, the decisions responding to some sensory signals can be made locally and immediately, once the sensory signals are perceived [6]. Localized processing can not only quickly respond to external stimuli and protect living creatures from further injury, but also reduce the computational burden of the brain [6,7]. This is considered a potential approach for designing and constructing a neuromorphic system by mimicking the structure and mechanism of its biological counterpart, which can realize both the biological neural-sensing and processing functions [8,9,10]. Therefore, in-memory computing and in-sensor computing have been proposed and the corresponding neuromorphic devices have been fabricated [11,12,13].

The emerging applications of neuromorphic systems, such as healthcare monitoring, neuro-prosthetics, and human–machine interfaces, require the flexibility of neuromorphic devices and systems in the morphology, which is highly related to the mechanical properties of the materials. Usually, bulk inorganic materials are rigid and incompatible with flexible electronics. In order to realize the high flexibility of the devices, several approaches have been proposed. The employment of organics, such as biomaterials and polymers, in the fabrication of the devices is one of the possible solutions [14,15,16,17,18]. Although devices with high flexibility can be obtained with organic materials, environmental sensitivity (e.g., temperature, moisture, and chemicals) is an issue influencing the long-term stability of the devices [19,20]. Inorganic materials also show high flexibility when the thickness of the materials is scaled to several micrometers and can be conformable with human skin, as reported in previous research. The use of inorganic materials is also a possible approach to fabricating flexible electronics [21,22]. 

Therefore, low-dimensional materials, including both 0-dimensional materials (0D), one-dimensional (1D) materials, and two-dimensional (2D), have been employed in flexible neuromorphic devices and systems [23,24,25,26,27]. In addition, some low-dimensional materials, such as transition-metal dichalcogenide (TMDs), graphene, and nanotubes, exhibit properties that are sensitive to external stimuli, which is also required for sensing applications. In addition, power consumption is also an issue that needs to be considered in flexible electronics for neuromorphic sensing applications. Thus, a number of self-powered flexible sensers were also designed and fabricated [28,29,30,31].

Therefore, energy efficient in-sensor computing devices, fabricated with low-dimensional materials, show great potential applications in neuromorphic engineering [32,33,34]. In this review, the electrical and mechanical properties of low-dimensional materials, as well as corresponding advantages in the fabrication of flexible electronic devices, will be introduced. Then, recent progress in the design and fabrication of flexible neuromorphic devices and systems with 0D, 1D, and 2D materials will be covered in detail. Next, the application of neuromorphic devices in artificial-intelligence, neuromorphic-computing, and artificial perception systems will be considered. Finally, the challenges and opportunities of neuromorphic devices and systems will be discussed.

## 2. Low-Dimensional Materials

**Figure 1:**
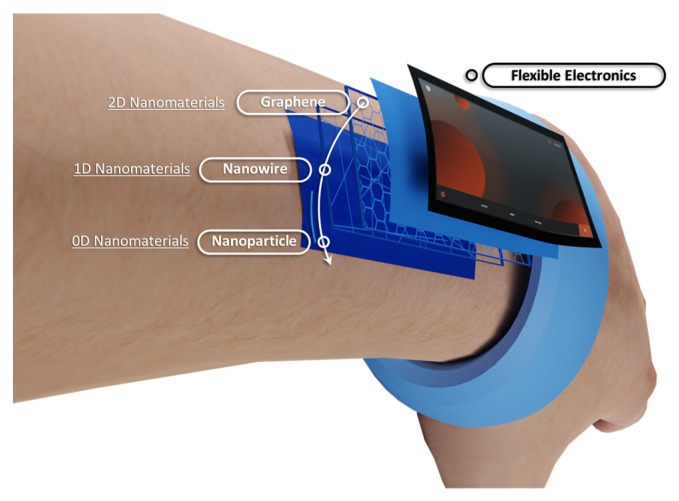
The application of low-dimensional materials in flexible electronics.

With the development of material science and processing technology, several novel materials with distinct electrical and physical properties have been discovered and synthesized. Among them, low-dimensional materials with at least one dimension at the nanoscale level, have distinct properties from their corresponding bulk materials [35]. Due to their outstanding properties, low-dimensional materials have attracted a great amount of attention [36,37,38,39,40]. For example, 2D materials, such as transitional metal dichalcogenides (TMDs), 2D oxides, and MXene, and 1D materials, such as carbon nanotubes, metal oxide nanotubes, and silicon nanowires, have been synthesized and widely applied. In addition, 0D materials, such as quantum dots and nanoparticles, were used in the dielectric layer of a memristive device [26,27,41]. These materials show superior flexibility for the implementation of flexible electronics than their bulk counterparts. Mechanical flexibility allows the devices to stably adhere on the surfaces of an arbitrary object, including human skin and organs, as shown in Figure 1. [42]. In addition, the electrical properties of low-dimensional materials range from insulators to conductors, and a number of flexible electronics, ranging from flexible electrodes to heterostrcure devices consisting of both insulators and conductors, can be fabricated with low-dimensional materials. Furthermore, the properties can be tuned by various methods, which will further contribute to the various applications of low-dimensional materials. For example, the electrical and physical properties of 2D materials can be tuned by adjusting the layer or doping with other elements [43]. For1D and 0D materials, the modification of the surface morphology and the functional groups can also change the properties [44,45]. In addition, size, diameter, and surface roughness will also lead to a variety of electrical and physical properties of low-dimensional materials [46,47].

Hence, in this section, we will provide a brief introduction to low-dimensional materials and their applications in flexible electronics.

Before discussing low-dimensional materials and their artificial synapses applications in detail, Table 1 provides a brief summary of the advantages and disadvantages of low-dimensional materials.

### 2.1. Zero Dimensional Materials

**Figure 2:**
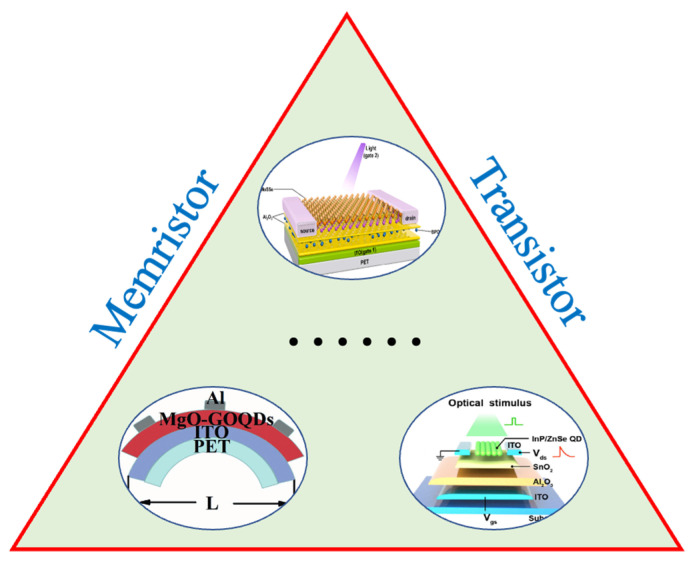
Representative 0D materials used in the fabrication of artificial synapses. The 0D materials can be used in both memristors and transistors incorporated with other materials [51,53,54]. Reprinted/adapted with permission from Ref. [51]. Reprinted/adapted with permission from Meng, 2021. Reprinted/adapted with permission from Ref. [53] Yu, 2021. Reprinted/adapted with permission from Ref. [54] Liang 2022.

0D materials are the group of materials that have all three dimensions less than 100 nm, such as nanoclusters, quantum dots (QDs), and nanoparticles [48]. Changes in spatial structure, morphology, size, and other parameters of 0D materials will lead to versatility in their physical and chemical properties [49]. Due to their unique electrical and optical properties and their tuning properties, 0D materials have been widely employed in various electronic devices, including flexible artificial synapses, as shown in Figure 2 [50,51,52].

The performance of both memristors and transistors can be enhanced with the outstanding properties of 0D materials [51,53,54]. For the application in memristors, conductive or semiconductive QDs were frequently used as the charge-trapping materials to increase uniformity in the performance of resistive switching among different cycles [55]. Chen reported a MgO-graphene oxide quantum dot hybrid film with a solution processed method. The device exhibited highly controllable RS behavior, due to the enhancement of the local electric field by QD and the redox of QD under an electric field, and the basic synaptic behavior could be emulated [53,56]. For the application in synaptic transistors, conductive or semiconductive QDs can be embedded in the charge trapping layer or the dielectric/semiconductor interface of the transistor to store the charges. As a result, the synaptic characteristics can be mimicked. For example, Meng reported a synaptic transistor with a 2D MoSe channel and an 0D BPQD trap layer. The device had low power consumption of 0.86 fJ/spike and was able to emulate the classical conditioning of Pavlov’s dog [51,55]. In addition to the improvement in the electrical performance, the optical properties of the materials could make the artificial synapse responsive to both electrical and optical signals, which contributed to the advancement of optoelectronic synapses for neuromorphic electronics and artificial intelligence.

### 2.2. One-Dimensional Materials

**Figure 3:**
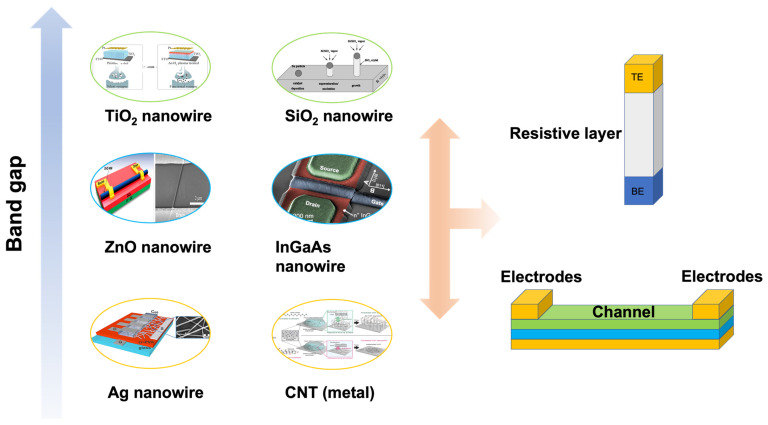
Representative 1D conductor, semiconductor, and insulator materials used in the fabrication of neuromorphic devices. The 1D materials can be used as a resistive layer of memristor, channeling materials of the transistor and electrodes of both types of devices [60,61,62,63,64,65]. Reprinted/adapted with permission from Ref. [60] Albano, 2017. Reprinted/adapted with permission from Ref. [61] Chen, 2016. Reprinted/adapted with permission from Ref. [62] Hong, 2008. Reprinted/adapted with permission from Ref. [63] Zota, 2016. Reprinted/adapted with permission from Ref. [64] Shan, 2020. Reprinted/adapted with permission from Ref. [65] Sun, 2003.

Conductors, semiconductors, and insulators are three basic categories of materials, classified on the basis of their bandgaps or conductivity, for the fabrication of electronic devices. Conventional bulk materials with outstanding electrical properties are less useful in flexible electronics, which is desired for wearable systems, due to their brittleness and rigidity. By contrast, 1D nanomaterials, a group of low-dimensional materials, have been widely studied, as shown in Figure 3. For instance, conductive and semiconductor nanowires are used for the electrodes and channels, respectively, of synaptic transistors [57]. The memristor can be fabricated with various nanowires [58,59].

Metal nanowires, such as Ag nanowire (AgNW), are typical conductive nanowires used in flexible electronics, due to their excellent mechanical deformability, high conductivity with less than 20 Ω/sq, and high transparency with a transmittance of 85% [60]. In addition to their employment in drain/source/gate electrodes of transistors, AgNW is also widely employed in flexible and wearable sensors [66]. Carbon nanotube (CNT), another typical nanowire, exhibits conductor and semiconductor properties, depending on its structure [61]. In particular, the conductor-type CNT shows high conductivity and excellent mechanical deformability, due to the high aspect ratio of its structural characteristics [67]. In addition, the electrochemical properties can be modified according to requirements through a wide range of functional groups. This phenomenon is beneficial for the fabrication of memristive devices, whose working mechanism is based on the trapping/detrapping of the carriers [23]. However, if high transparency is also required, the layer of the conduction network should be carefully controlled, as a monolayer or a few layers [68]. In addition, metal nanowires can be used in memristors when they are decorated with other materials, such as some polymers [69].

Semiconductor-type nanowire is an indispensable component in flexible electronics, especially for transistor-based devices. Conventional semiconductor-based nanowires, such as silicon, germanium, and other compound semiconductor nanowires, have been widely studied, due to their excellent chemical stability, optical properties, and compatability with complementary metal oxide semiconductor (CMOS) technology. Kim et al. reported on an InGaAs nanowires-based field effect transistor (FET), which shows high stability and low variation in threshold voltage shift [63]. Apart from conventional semiconductor nanowires, metal-oxide nanowires have attracted great attention due to the convenient synthesis method [63,70]. As previously reported, most metal-oxide nanowires can be synthesized by either hydrothermal or chemical vapor deposition (CVD) methods [71,72,73]. For example, Hong et al. synthesized zinc oxide (ZnO) nanowires by the CVD method and fabricated the ZnO-based FETs by transferring the nanowire to a pre-prepared substrate [62]. Both enhancement- and depletion-mode transistors were fabricated by tuning the diameter of the ZnO nanowires. Hence, the fabrication of flexible transistors with 1D materials provides a possible solution for the construction of transistor-type artificial synapses and corresponding neuromorphic systems [16]. Some semiconductor nanowires, such as Si nanowire and ZnO nanowire, can also be used as resistive layers of memristor devices [74,75].

Insulator nanowires, synthesized on the basis of metal-oxide materials, are widely used in memristor devices due to the wide bandgap, although semiconductor nanowires and decorated metal nanowires can also be used as the resistive layer of a memristor [58]. Compared with conventional memristors that are based on bulk metal-oxide insulators requiring a high working voltage and exhibiting rigidity in morphology, flexible memristors with a low working voltage can be fabricated with nanowire materials. Sun reported on the SiO_2_ nanowire with a soft break phenomenon, indicating that nanowires can be used to fabricate memristor devices [65]. Shan investigated a vertical-structure memristor based on TiO_2_ nanowires, which implied that the fundamental synaptic characteristic can be emulated [64].

### 2.3. Two-Dimensional Materials

**Figure 4:**
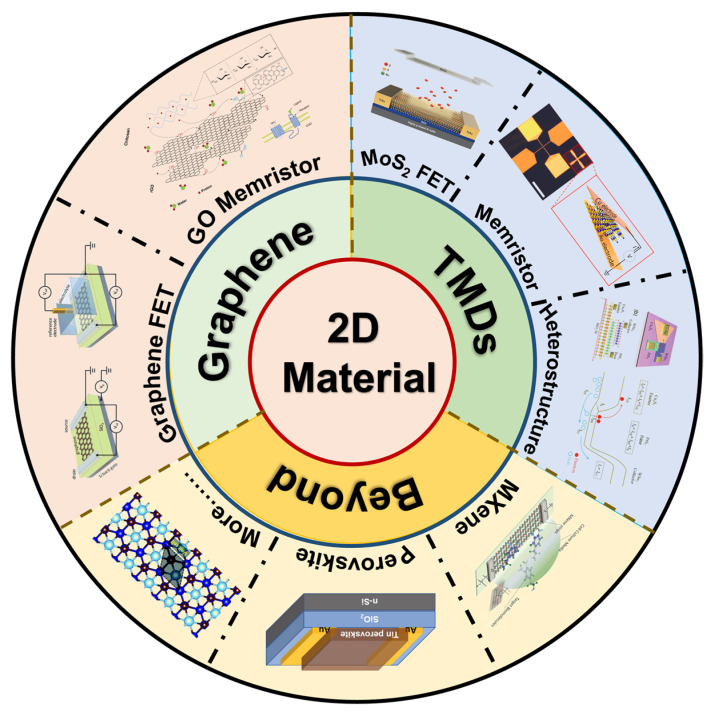
Representative 2D materials used in the fabrication of flexible artificial synapses; 2D materials are frequently used as the channel materials of transistor and resistive layers of memristors [23,77,78,79,80,81,82,83]. Reprinted/adapted with permission from Ref. [23] Lu, 2020. Reprinted/adapted with permission from Ref. [77] Fu, 2017. Reprinted/adapted with permission from Ref. [78] Liu, 2017. Reprinted/adapted with permission from Ref. [79] Xu, 2019. Reprinted/adapted with permission from Ref. [80] Yang, 2021. Reprinted/adapted with permission from Ref. [81] Xu, 2016. Reprinted/adapted with permission from Ref. [82] He 2019. Reprinted/adapted with permission from Ref. [83] Shao 2021.

Since the thickness of the material is one of the dominant factors for the flexibility of the devices, 2D materials with one or a few layers of atoms have a great advantage in the fabrication of flexible devices [76]. Therefore, a number of 2D materials have been employed in the design and fabrication of flexible devices, based on their properties, as shown in Figure 4.

Graphene, a carbon allotrope composed of one layer of carbon atoms, was the first reported 2D material [37]. Although graphene transistors’ low on/off ratio, attributed to its gapless band structure, hinders its applications in digital devices, its high carrier mobility of up to 250,000 cm^2^ V^−1^ s^−1^ contributes to its application in analog devices and electrochemical sensors [84]. In addition, its mechanical flexibility and outstanding Young’s modulus, due to strong atomic bonding, provide a foundation for the fabrication of flexible electronics [77]. In addition to graphene, graphene oxide (GO) shows a number of advantages in memristive devices and chemical sensors, due to the plethora of functional groups [23].

TMDs, a group of 2D materials with a non-zero bandgap, obtain tunable electrical and physical properties by various methods, such as defect engineering, electrostatic doping, and chemical intercalation [43,85,86], which contribute to modifying the performance of FETs when TMDs are employed as the channel materials. MoS_2_, a representative 2D material with high mobility and a suitable bandgap, is one of the most widely studied 2D materials recently. For example, MoS_2_ FETs with low-threshold voltage shift, free hysteresis, and long-term reliability under bias were reported, involving the dielectrics’ optimal deposition and passivation methods [78]. Moreover, benefiting from the atomic thickness of 2D materials, memristors with ultra-low voltage at several hundred millivoltages, called atomristors, can be achieved [79,87]. Although the underlying mechanism for the memristive behavior is unclear, this device provides a novel approach to constructing low-power neuromorphic devices. Taking advantage of the atomic thin property and free-standing nature of the 2D materials, a wide range of van der Waals heterostructures can be obtained by stacking various individual layers on top of each other without the constraints of lattice matching and processing compatibility. The heterostructure shows superior performances in optical, electrical, and electrochemical properties than those of single 2D materials. Yang reported a Cu_9_S_5_/PtS_2_/WSe_2_ double-heterojunction bipolar transistor with an excellent current gain (β ≈ 910) [80]. Kim studied a heterojunction FET by engineering the band structure, and a low threshold swing of about 22 mV/dec was obtained, which paved the way for applications in low-power devices [88].

In addition to conventional carbon-based 2D materials and TMDs, some novel 2D materials, such as MXene and perovskite, have been reported and employed in flexible electronics. For example, benefiting from its excellent mechanical, electrical, chemical, and physical properties and its hydrophilic surface, MXene has been widely used in energy storage, nanocomposite fabrication, and chemical sensing [89,90,91]. Xu fabricated a label-free MXene-FET using ultrathin-conductive Ti_3_C_2_-MXene micropatterns for detecting dopamine, and a temporal resolution of ≈50 ms for neural activity was obtained [81]. In addition, perovskite and other novel 2D materials were investigated in the application of electronic devices, although the long-term stability in ambient conditions still needs further improvement [82,83].

## 3. Flexible Artificial Synapses

### 3.1. Biological Synapses

**Figure 5:**
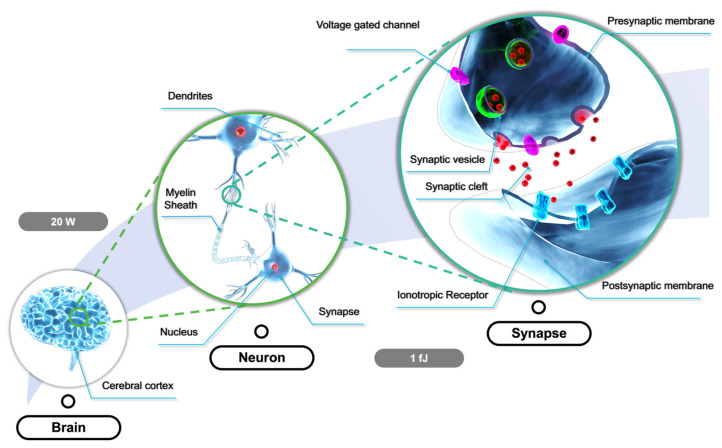
The schematic diagram for biological synapses, which consist of the presynaptic membrane, the synaptic cleft, and the postsynaptic membrane.

In the human brain, there are 10^11^ neurons and 10^15^ synapses that are the basic components for physiological signal transmission and processing [92]. As shown in Figure 5, the biological synapses consist of the presynaptic membrane, the synaptic cleft, and the postsynaptic membrane. Signals propagate from the axons of one neuron to the dendrites of another neuron via synapses and the functional connection. Moreover, the strength of the connections between the presynaptic and postsynaptic neurons plays a significant role in information processing [93]. The signal transmission begins with the opening of calcium channels in the presynaptic membrane, which is stimulated by nerve impulses. Then, the vesicles gradually move to the presynaptic membrane, where neurotransmitters in the vesicles are released into the synaptic cleft and bind to receptors on the postsynaptic membrane. Finally, the chemically gated ion channels coupled to the receptors open, allowing the entry of relevant ions into the postsynaptic cells [94]. Short-term plasticity (STP), long-term plasticity (LTP), and spike-rate-dependent plasticity (SRDP) can be observed, depending on the stimuli. This process consumes only approximately 1–10 fJ. As a result, the power of the human brain is around 20 W, which is significantly lower than the that of the existing artificial intelligence computing system [95]. Therefore, mimicking the structure and working principles of biological counterparts is a potential approach in fabricating artificial synapses, which are the basic blocks of artificial neural networks [96,97].

### 3.2. Memristor-Type Artificial Synapse

**Figure 6:**
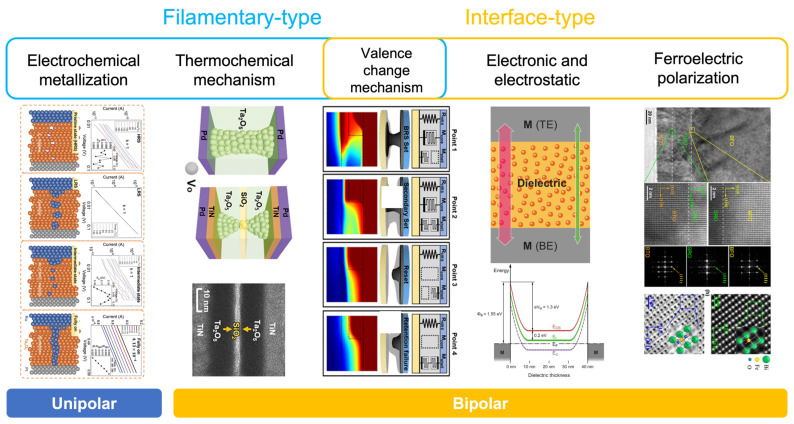
The typical working mechanism of memristors. Filamentary-type and interface-type are the two main categories [99,100,101,102,103]. Reprinted/adapted with permission from Ref. [99] Yoon, 2020. Reprinted/adapted with permission from Ref. [100] Xu, 2019. Reprinted/adapted with permission from Ref. [101] Park 2022. Reprinted/adapted with permission from Ref. [102] Schroeder, 2010. Reprinted/adapted with permission from Ref. [103] Sun, 2020.

In this section, we focus on the discussion of the memristor-type artificial synapse in terms of its structure and working mechanism. A memristor, typically consisting of two metal plates with a switching layer sandwiched between them, is formalized as a circuit element, based on the concept of resistance switching that was developed in 1971 [98]. Resistance switching is a reversible process, in which a device changes its resistance in response to external stimuli, such as electricity or light, which leads to either non-volatile or volatile states. Therefore, the memristor is considered to be one of the prototypes for artificial synapses. In addition, benefiting from a simple structure, a high-density matrix of devices can be fabricated. As a result, the memristor-type artificial synapse is regarded as a potential candidate for the neuromorphic system. Although various materials have been employed in the fabrication of memristor devices, most of the memristors work on the basis of the working principles shown in Figure 6.

Electrochemical metallization (ECM) and thermochemical mechanism are two typical working principles of the filamentary-type memristor. Since the filamentary-type memristor typically exhibits a non-volatile behavior and has good retention properties, it shows great potential for applications in neuromorphic computing. For an ECM memristor, the top plate is usually made of active metal materials, such as Cu and Ag, while the bottom plate is made of inert metal materials, such as Pt and Au. The redox effect that occurs under voltage bias leads to the formation/disruption of the conduction path between the two plates, and the device can be switched between a high-resistance state (HRS) and a low-resistance state (LRS). Hirose reported this phenomenon in 1976, and Yang proposed the ECM model after a series of studies on this topic [104,105,106]. A memristor based on the thermochemical mechanism is usually a unipolar device, and the generation of Joule heat is the dominant operating mechanism for resistance switching. Briefly, the Joule heat generated by the current changes the local chemical composition, changing the stoichiometric ratio or forming an oxygen vacancy. Therefore, the switching behavior of the resistance can be observed [107,108]. Taken the Au/Cu_3_(BTC)_2_/Au structure device as an example for detailed discussion, the reaction of Cu^+^ with the BTC linker is a cause of the change in the resistive state [109]. When the high electric field is applied onto the Au/Cu_3_(BTC)_2_/Au device, Cu^2+^ ions at the localized crystalline defects can be dislocated from the BTC linkers, driven into the top Au layer, and reduced to Cu atoms. The negatively charged vacancies in the Cu_3_(BTC)_2_ are relatively less stable, and the carboxylic groups can be removed from the aromatic linkers upon Joule heating and emitted as carbon dioxide through the top electrodes. The pyrolysis of the linkers may then lead to the coupling of the neighboring benzene rings and the subsequent formation of sp2-hybridized carbon-rich channels.

A memristor based on a valence-change mechanism (VCM) can be either filamentary-type or interface-type, depending on the properties of the resistive materials. For example, Kwon reported a VCM-based filamentary-type memristor with a TiO_2_ dielectric. Based on the in situ physical and electrical measurement, the switching behavior was attributed to the formation and disruption of Ti_n_O_2n−1_ (Magnéli phase), due to the migration of the oxygen vacancy under voltage bias [110]. Li fabricated an interface-type memristor with an Au/MoS_2_/Au structure. Due to the redistribution of the vacancies and the modulation of the semiconductor/metal Schottky barrier under voltage bias, a transition between rectification-mediated and conductance-mediated behavior in the voltage-current characteristics can be observed [111].

Compared with the filamentary-type memristor, the interface-type memristor usually has a gradual change in the resistance state, rather than an abrupt change [112]. However, the interface-type device based on the modification of the interface barrier usually exhibits volatile behavior, which is not desired for the neuromorphic computing system. Fortunately, recent examination of these devices indicated that the volatile memristor can be applied in constructing an artificial sensory system. Wang reported a purely electronic memristor with a Ti/ZnO/Pt structure. The quasi-Ohmic contact at the ZnO/Pt interface and the Schottky contact at the Ti/ZnO interface played a significant role in the device’s performance [113]. Ferroelectric devices are another interface-type memristor that works on the basis of the ferroelectric polarization property of the ferroelectric dielectrics. With this working mechanism, the ferroelectric device is able to overcome the drawbacks observed in the filamentary-type device, such as the poor cycle-to-cycle variation and the limited number of conduction states. Li reported a memristor based on 2D ferroelectric CuInP_2_S_6_ dielectrics, which has a large on/off ratio larger than 6 × 10^3^ and small set and reset voltage variations of 5.3% and 9.1%, respectively [114].

### 3.3. Synaptic Transistor

**Figure 7:**
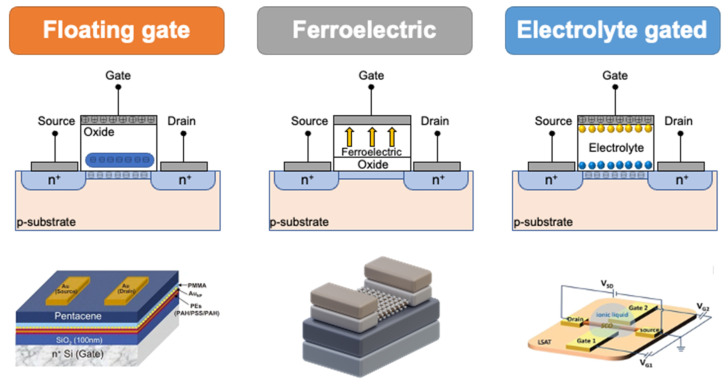
Schematic diagram for the three typical prototypes for the synaptic transistors, floating-gate, ferroelectric, and electrolyte-gated transistors [115,116,117,118,119,120]. Reprinted/adapted with permission from Ref. [115] Huang, 2019. Reprinted/adapted with permission from Ref. [116] Van, 2016. Reprinted/adapted with permission from Ref. [117] Si, 2019. Reprinted/adapted with permission from Ref. [118] Ilić, 2020. Reprinted/adapted with permission from Ref. [119] Qi, 2015. Reprinted/adapted with permission from Ref. [120] Huang, 2021.

In addition to the two terminal artificial synapses based on the memristor, another basic semiconductor device, a transistor, can also be used as the prototype for artificial synapses, especially for multi-terminal devices. Due to its multi-terminal structure, this type of artificial synapse can be integrated with the sensing unit and contribute to the realization of in-sensory computing, which will be beneficial in the development of an artificial sensory system. Floating-gate transistors, ferroelectric transistors, and electrolyte-gated transistors are the three main categories for the construction of synaptic transistors, as shown in Figure 7.

The floating-gate transistor, whose dielectric layer consists of a tunneling layer, a charge trapping layer, and a block layer, is widely employed as the basic structure of non-volatile memory [118]. Compared to the transistor with a single oxide dielectric layer, the charges can be stored in the charge-trapping layer without the need for any applied voltage [121]. However, the conventional structure has challenges in device scaling and cell-to-cell interference. Therefore, the nanocrystals were used to replace the oxide-based charge-trapping layer. In addition, with the employment of the nanocrystals as the charge-trapping layer, more conductance states can be achieved, which is beneficial for the performance of artificial synapses. However, floating-gate memory usually requires a high voltage to inject the charges in the charge-trapping layer, and the high power consumption is still an issue to be addressed when it is applied in neuromorphic computing systems.

In addition to the floating-gate transistor, the ferroelectric transistor was also used for non-volatile memory. In contrast to the charge-trapping memory, the ferroelectric device has a similar but simple structure, as shown in Figure 7 Recently, HfO_2_-based ferroelectrics, organic polymer ferroelectrics, and 2D ferroelectrics have been employed as the dielectric materials of transistors. Ascribed to the CMOS compatibility, HfO_2_-based ferroelectric shows great potential in large-scale integration and has attracted extensive research interest [119,122]. The polymer-based ferroelectric material shows high flexibility and can be prepared with low-cost solution process methods. Therefore, it is highly preferred in the fabrication of wearable electronics [123,124]. The 2D ferroelectric material, a novel material with atomic thickness, has also been used to fabricate ferroelectric transistors, and the formation of a heterostructure to realize some unique properties is one of the advantages of applications of 2D materials [125,126]. However, as an important parameter for neuromorphic computing, retention is a key problem to be solved for ferroelectric transistors [127].

As mentioned above, both STP and LTP are significant features that can be observed in biological synapses. However, floating-gate and ferroelectric transistors usually only exhibit LTP behavior in response to electrical stimuli. Electrolyte-gated transistors, which work based on either an electric double layer (EDL) or electrochemical doping, can exhibit both STP and LTP in a single device [120,128]. In addition, due to the EDL effect, a huge capacitance can be observed at a low voltage, and the transistor is able to work at a voltage smaller than 1 V, which opens a new avenue for the design and fabrication of low-power artificial synapses. Moreover, since electrolyte can be deposited and patterned with solution-processing methods, such as inject printing or screen printing, it will be compatible with flexible electronics [129,130].

## 4. Neuromorphic System

Artificial synapses can be used in the construction of neuromorphic computing and artificial perception systems based on the performance of the devices. Both memristor- and transistor-type artificial synapses can be used in neuromorphic computing and artificial perception systems. Usually, LTP is the foundation for neuromorphic computing, and the memristor device shows a great advantage in terms of large-scale integration for the artificial neural network, due to its crossbar structure [131]. With regard to the artificial perception system, STP is required, and synaptic transistors with multi-terminal structures are preferred [132]. Meanwhile, by using functional materials, transistor-type artificial synapses sensitive to non-electrical stimuli can be designed, which will further promote their applications in artificial perception systems, such as visional systems and tactile systems.

### 4.1. Neuromorphic Computing

#### 4.1.1. Artificial Synapses with 0D Materials

**Figure 8:**
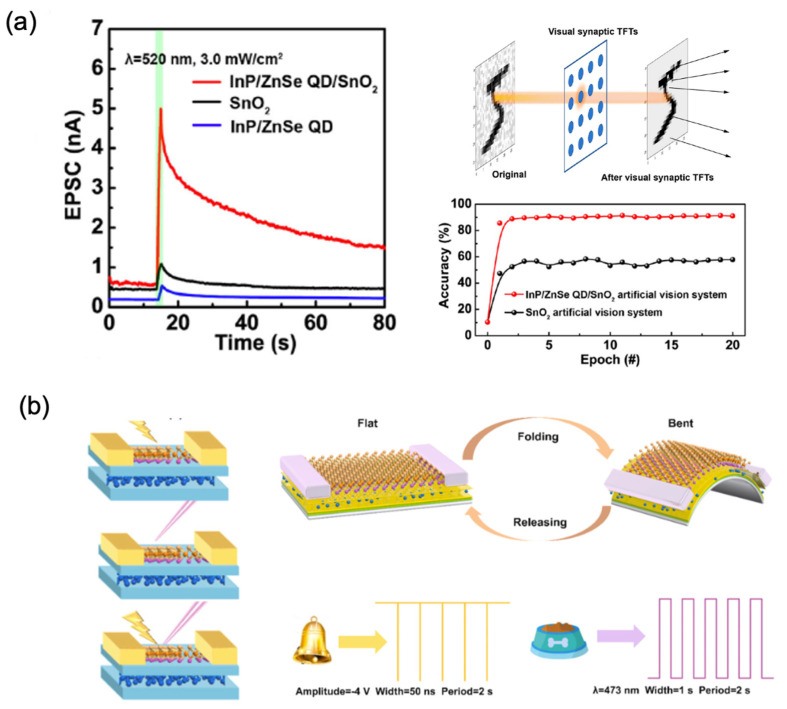
(**a**) The synaptic behavior of the InP/ZnSe QD/SnO_2_ structure artificial synapse discussed in Figure 2 and the image-recognition rate after training with InP/ZnSe QD/SnO_2_ and base SnO_2_ synaptic TFTs, based on an artificial-vision system [51] Reprinted/adapted with permission from Ref. [51] Meng, 2021. (**b**) Schematic diagram of different modulations of a 2D MoSe/0D BPQDs-based artificial synapse, the bending of the device, and the simulation of classical conditioning of the Pavlov’s dog experiment [54] Reprinted/adapted with permission from Ref. [54] Liang, 2022.

As discussed above, with their unique electrical and optical properties, 0D-material-based artificial synapses show great potential in the fabrication of optosynapse and optical-based neuromorphic computing and neuromorphic systems. Figure 8a shows the change in the excitatory postsynaptic current (EPSC) of SnO_2_, InP/ZnSe QDs and InP/ZnSe QD/SnO_2_ hybrid TFTs under a presynaptic optical pulse (3 mW/cm^2^, 2.5 s) at a wavelength of 520 nm. The basic synaptic behavior can be observed and simultaneous sensing, low-level preprocessing, and image denoising were simulated, based on the synaptic characteristics of the device. A digital number in the Modified National Institute of Standards and Technology (MNIST) database was used to study the device’s recognition accuracy, and a high accuracy of 91% was realized. The result indicates that this device shows potential in optoelectronic synapses for neuromorphic electronics and artificial intelligence [54].

Figure 8b shows the schematic diagram of different modulations of a 2D MoSe/0D BPQDs-based artificial synapse. The artificial synapse had different responses to voltage-only, optical-only, and mixed photoelectric stimulations. MoSe possesses a distinguished photoresponse in the UV range and, hence, there was a rapid generation of charge carriers. As a result, synergistic accumulation or subtraction can be realized with the assistance of voltage pulses. Based on the dual response of the device to optical and electrical signals, the device was used to simulate the classical Pavlovian dog conditioning experiment, which is of great significance for the study of neuromorphic behavior [51].

#### 4.1.2. Artificial Synapse with 1D Materials

**Figure 9:**
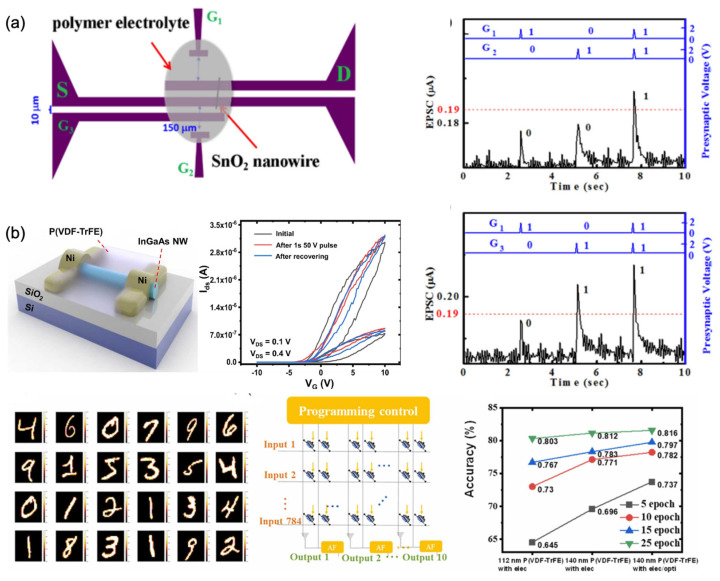
(**a**) Schematic diagram of a multi-gate SnO_2_ transistor covered with polymer-electrolyte and its application in logic gate [57] Reprinted/adapted with permission from Ref. [57] Zou, 2017. (**b**) Schematic illustration of the synaptic device structure, transfer characteristic curves of the device, and its application in supervised learning on the MNIST database [133] Reprinted/adapted with permission from Ref. [133] Xie, 2022.

As a representative group of low-dimensional material, 1D materials have also been used in the design and fabrication of artificial synapses. Figure 9a shows a schematic diagram of the polymer-electrolyte-gated nanowire transistor used to measure the synaptic logic function. The EPSC values of G1 and G2 were measured to be almost identical when a presynaptic spike was applied on either G1 or G2. When spikes applied at G1 and G2 were triggered simultaneously, the amplitude of the EPSC increased. When a spike is applied on the G3 terminal with a larger size and close to the channel, a different EPSC was observed than that of G1. Based on this result, the logic functions ‘‘AND’’ and “YES G3” could be realized [57].

Figure 9b shows the schematic diagram for the ferroelectric P(VDF-TrFE)-wrapped InGaAs NW artificial synaptic devices. Changes of hysteresis windows attributed to the charge trapping by native oxides and surface defect states were observed in the transfer curves. The synaptic characteristics can be realized and a hardware neural network was simulated for supervised learning on the MNIST database. Accuracy of training of over 80% was obtained. These results indicated that the 1D material has potential in the application of neuromorphic computing [133].

#### 4.1.3. Artificial Synapses with 2D Materials

**Figure 10:**
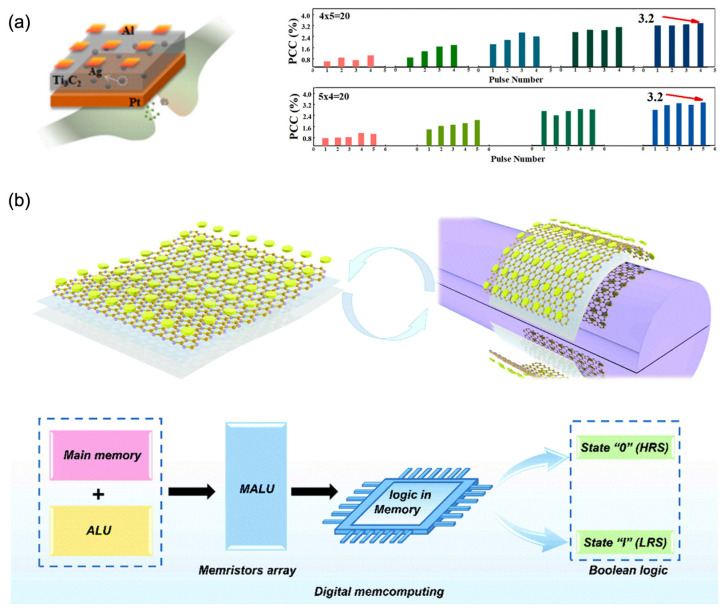
(**a**) The schematic diagram for the memristor-type artificial synapse with Al/Ti_3_C_2_: Ag/Pt structure and the percent changes of the device current (PCC) [97] Reprinted/adapted with permission from Ref. [97] Tang, 2019. (**b**) Mechanical flexibility of the proposed memristor and the schematic diagram for the logic memristor array acting as memory arithmetic logic unit (MALU) with the merged functions of memory and ALU [98] Reprinted/adapted with permission from Ref. [98] Tang, Yoon, 2020.

This paragraph concentrates on the recent advances in memristors with 2D materials for neuromorphic computing applications. Figure 10a shows a memristor matrix with Ag-nanoparticledoped Ti_3_C_2_, as the resistive layer. The basic synaptic behaviors, such as EPSC, STP, and LTP, can be emulated successfully. Moreover, energy consumption as low as 350 fJ/spike can be obtained. The microstructure and finite-element analysis (FEA) indicate that the filaments formed by interaction between the atomic vacancies and metal ions dominate the working mechanism of the device. As a proof of concept, decimal arithmetic operations, such as addition and multiplication, were implemented successfully, promoting the diversification development of 2D materials in the field of neuromorphic chips [134].

Boron nitride, another 2D material with atomic thickness, is considered as a candidate for flexible memristors. Figure 10b presents a flexible memristor based on 2D boron nitride for low-power consumption in-memory computing [135].The flexible BN-based memristor exhibited stable bipolar resistance-switching behavior, ultralow power consumption of 198 fJ/spike, excellent switching endurance of more than 10^4^, and reliable retention characteristics longer than 10^5^ s. In addition, the basic synaptic characteristics, such as STP, pair pulse facilitation (PPF), LTP/long-term depression (LTD), and STDP, were emulated successfully. As a demonstration, Boolean logic gates, such as FALSE, IMP, and NAND, were implemented with the memristor matrix. This result indicated that this device shows great potential for implementing an ultrafast, ultra-low-power consumption and high-efficient in-memory computing intelligent wearable system.

### 4.2. Artificial Perception Systems

The first artificial perception system, which consisted of a pressure sensor, an oscillator, and a synaptic transistor, was reported by Bao’s research group in 2018 [136]. In that research work, the synaptic transistor was used to process the signals received and converted by the pressure and oscillator, respectively. The signal modulated by the synaptic transistor was compatible with the biological signal and showed a potential to work as the interface between an artificial system and a biological system. To date, a great amount of effort has been put into this area to construct the artificial perception systems based on various structures [136,137,138]. Among the various artificial synapses, the synaptic transistor has a great advantage in the construction of artificial perception systems, due to its multi-terminal structure, and showed the possibility of employing the functional materials as the active layer to realize in-sensor computing, as discussed above. Therefore, in this section, we mainly concentrate on the development of the artificial perception systems with an artificial synapse as the signal-processing unit.

#### 4.2.1. Artificial Perception Systems with 0D-Material-Based Artificial Synapses

**Figure 11:**
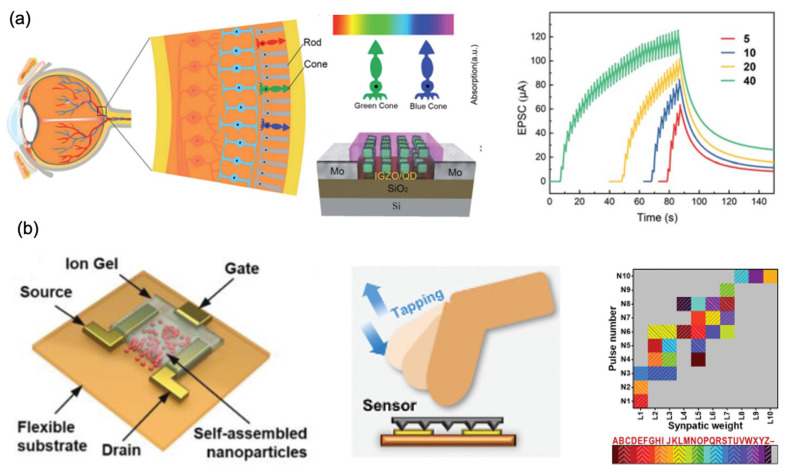
(**a**) The schematic diagram for the human visual system, consisting of rods and three types of cones, red, green, and blue. Learning and memorizing capability of this photonic synaptic device under stimulated light (450 nm) accorded with the various pulse numbers and pulse times [139] Reprinted/adapted with permission from Ref. [139] Xin, 2021. (**b**) The structure of ion-gel-gated synaptic transistor composed of interdigitated electrodes, a self-assembled NP channel, and chitosan-based electrolyte on a polyimide flexible substrate. Recognition of tactile patterns took place by tapping Morse code and the classification of 26 letters on a 10 × 10 mapping [140] Reprinted/adapted with permission from Ref. [140] Jiang, 2022.

This section discusses the construction of an artificial perception system with 0D-material-based artificial synapses. Figure 11a shows the schematic diagram of the human visual system and the rods and the three type of cones. The cones and rods are sensitive to color and light intensity, respectively, and work as the color signal input. Color perception is determined by the human brain. The learning and memorizing capability of the photonic synaptic device under stimulated light was also demonstrated. The increase in the number of pulses or the pulse duration led to EPSC enhancement and maintained a certain conductivity or synaptic weight. In biology, this is referred to as sensing, learning, and memory for different colors [139].

Figure 11b shows the ion-gel-gated synaptic transistor composed of interdigitated electrodes, a self-assembled nanoparticles (NPs) channel, and chitosan-based electrolyte on a polyimide flexible substrate. The uniform and defect-less inorganic metal oxide NPs were used as the semiconducting material for building the synaptic device. A good interface quality between the ion gel and the channel, which is beneficial for the stability of the device, and high surface/volume ratio of the NPs were observed. The synapse characteristics were measured from the device. As a proof of concept, recognition of tactile patterns by tapping Morse code, which were generated by manually controlling the tapping movement of a finger, was implemented. The classification of 26 letters on a 10 × 10 mapping with an accuracy of 94% was realized [140].

#### 4.2.2. Artificial Perception Systems with 1D-Material-Based Artificial Synapses

**Figure 12:**
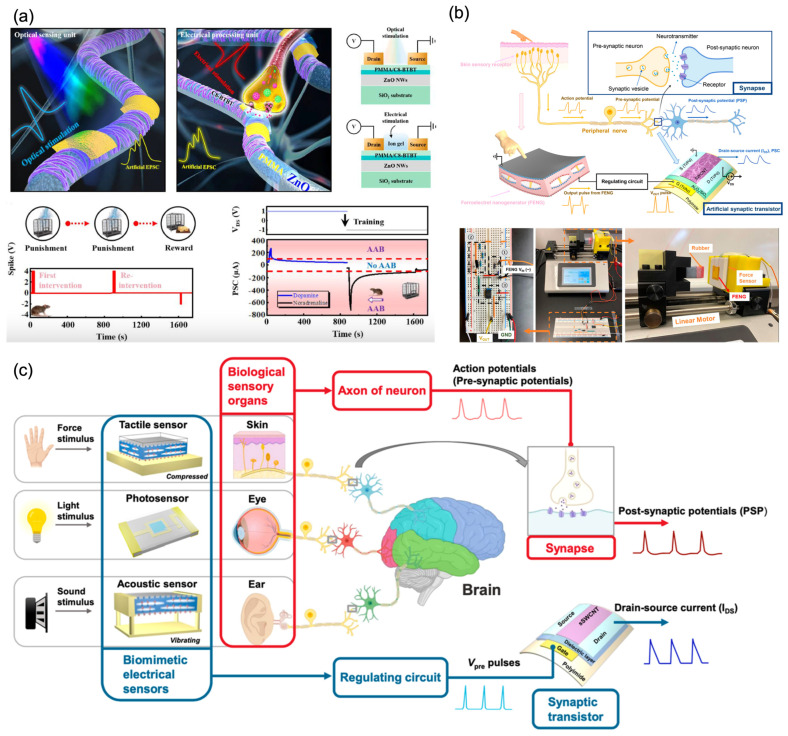
(**a**) A photoelectric neuromorphic transistor composed of ZnO nanowire with a PMMA/C8-BTBT decorated sheath [105]. Reprinted/adapted with permission from Ref. [105] Yang, 2012 (**b**) Schematic illustration of biological skin and synapse, in comparison with their artificial electrical counterpart [106]. Reprinted/adapted with permission from Ref. [106] Yang, 2014 (**c**) Schematic illustration of biological visual, auditory, and tactile sensory organs and the process of information transmission and storage through neuron and synapse [107] Reprinted/adapted with permission from Ref. [107] Yang 2009.

Figure 12a presents the synaptic transistor with 1D ZnO wire as the channel material [141]. Due to the optoelectronic properties, the device is able to sense the optical stimuli with low intensity at the order of microwatts per square centimeter and process the signals as an electronic signal. A transition between STP and LTP can be obtained, corresponding to the stimuli. As a demonstration, the Skinner Box was implemented for the first time for pain-avoidance and pleasure-induction patterns. The results suggest that the device can be applied to control and design multipurpose neuromorphic systems by combining brain-like processing and artificial sensory nerves.

With the multi-terminal structure of the synaptic transistor, the connection of the sensing unit to the gate terminals of the transistor provides an alternative solution for constructing artificial perception systems. Figure 12b shows the schematic diagram for neuromorphic electronic skin and the biological counterpart. The ferroelectret nanogenerators and carbon nanotube-based flexible synaptic transistors were employed as the sensing and processing units, respectively, of the biomimetic electronic sensory skin. The synaptic characteristics were studied systematically in the research. The artificial system was able to imitate the mechanoreceptors and peripheral nerves for transducing and relaying the force stimulus information to the synapses. The results showed that the neurological electronic skin, which is expected to be used to interface with skeletal muscle fibers for applications in neuroprosthetic devices, closely mimics the behavior of actual human skin.

The biological sensory system is usually able to respond to various surrounding stimuli, such as visual, auditory, and olfactory signals, to extend its adaptability to the environment and avoid potential hazards. Figure 12c shows an artificial multimodal sensory–memory system based on the flexible semiconducting single-wall carbon nanotube (sSWCNT) synaptic transistor sensors. This system exhibits bioreceptor-like sensory capability and synaptic-like memory functions, such as touch-, hearing-, and vision-sensing abilities. In brief, the external stimuli were converted and regulated to electrical pulses, which were then sent into the artificial nervous system, represented in the research work by the flexible synaptic thin-film transistors. In addition, on the basis of the sensory–memory system, it was possible to simulate the well-known model of human memory and learning, which describes the transition of memory states from short-term to long-term memory through rehearsal. The result indicated that this artificial multimodal sensory–memory system, which is capable of imitating multiplex and multifunctional biological sensory systems, will contribute to the construction of environment-interactive artificial intelligence.

#### 4.2.3. Artificial Perception Systems with 2D Material-Based Artificial Synapse

**Figure 13:**
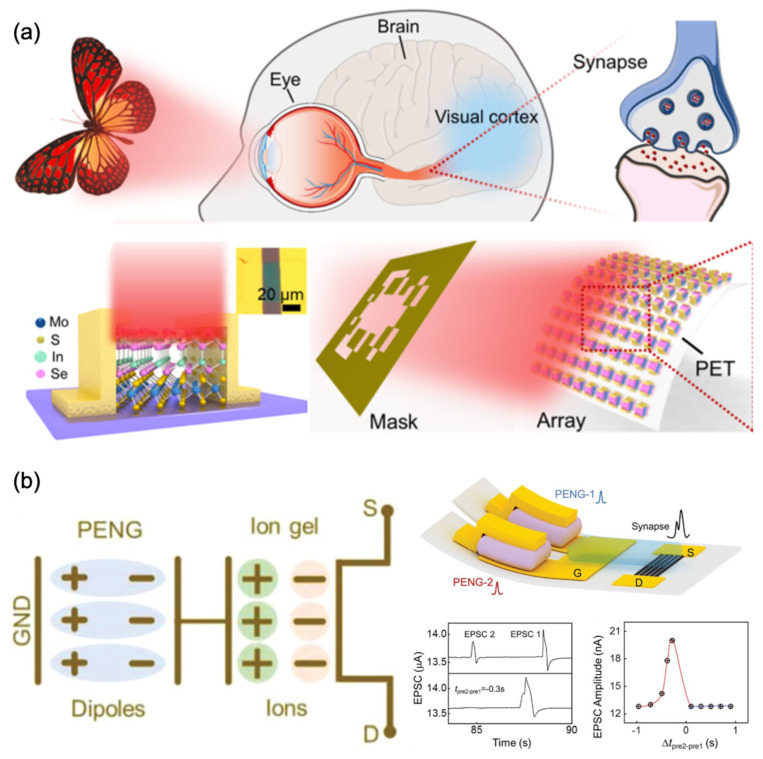
(**a**) Schematic diagrams for the biological visual system and the In_2_Se_3_/MoS_2_ synaptic device and the demonstration of imaging functions of the In_2_Se_3_/MoS_2_ synaptic device array [144] Reprinted/adapted with permission from Ref. [144] Hu, 2022. (**b**) Equivalent schematic diagram of the coupling effect of piezoelectric potential through an ion gel; the schematic illustration of the piezotronic graphene artificial sensory synapse with two artificial presynaptic sensor neurons (PENG-1 and PENG-2) and the EPSC response under various stimuli [146] Reprinted/adapted with permission from Ref. [146] Chen, 2019.

The 2D materials, as promising candidates for the fabrication of artificial synapses with a sensory function, due to their capacity to respond to external stimuli, have also been widely investigated. Figure 13a shows the schematic diagram of the human visual system detecting “butterfly” patterns. In brief, the retina of the human eyes can convert the external optical signals into electrical signals and transmit them to the brain for storage and processing [142,143]. In the reported work, the In_2_Se_3_/MoS_2_ heterostructure device was used to simulate the functions of optical synapses. There are Schottky barriers between the Au electrode and In_2_Se_3_/MoS_2_, hindering the transportation of photogenerated carriers and prolonging the photoresponse speed, which leads to the synaptic behavior of the devices. As a demonstration of a visual sensory system, the 10 × 10 In_2_Se_3_/MoS_2_ device array was illuminated by the NIR light through a butterfly-pattern mask and the optical signals were successfully sensed, process, and stored, which indicated that this device can mimic the biological visual functions [144].

Figure 13b shows a schematic illustration of the piezotronic artificial sensory synapse, which consists of a PENG as the power/sensation component and an ion-gel-gated graphene FET as the artificial synapse. The transmission process is realized by coupling the PENG piezopotential, due to mechanical strain, to the FET device through the ion gel. The piezopotential triggered by the external strain is the presynaptic stimulus in the piezotronic artificial sensory synapse, which is similar to the electric current transmitted between neurons in the human somatosensory system. The output signals between source and drain electrodes are considered as the postsynaptic current. The long-range polarization of cations/anions in the ion gel is used for the modulation of the charge carriers in the graphene channel [145]. With the artificial sensory synapse, the spatiotemporal distinguishing function was realized by the correlated stimulation from different presynaptic terminals, PENG-1 and PENG-2, to synergistically trigger one postsynaptic current. This demonstration indicated that the proposed piezotronic artificial sensory system is fundamental in constructing more complex and multifunctional artificial neural networks [146].

## 5. Conclusions and Perspectives

**Figure 14:**
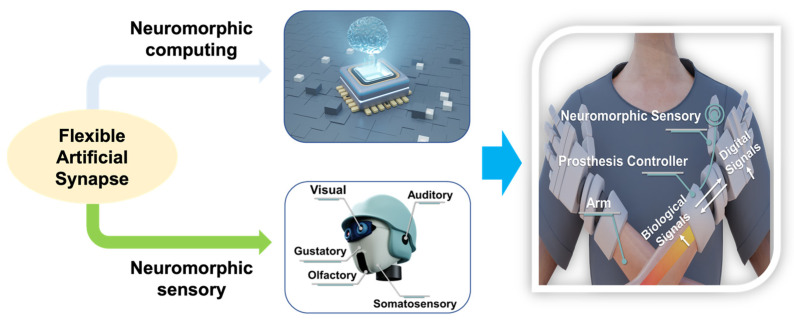
Example of the application of flexible artificial synapse in neuromorphic systems.

In this review, recent progress in flexible artificial synapses, which potentially revolutionized neuromorphic-computing and artificial perception systems, was discussed. Owing to their outstanding electrical and mechanical properties, such as tunable dielectric properties, hetero-integration compatibility, and high flexibility, low-dimensional materials have been considered as promising candidates in flexible artificial synaptic devices. In brief, 0D materials have unique electrical and optical properties. They can be used to enhance the performance of optoelectronic artificial synapses and have been widely used for optical signal processing and the construction of artificial vision systems. Conductive and semiconductive 1D materials can be used for the electrodes and channels of synaptic transistors, respectively. Memristor can be fabricated by various 1D nanowires themselves or by incorporating ID nanowires with other materials, such as polymer. Based on the synaptic characteristics of flexible artificial synapses with low-dimensional materials, as discussed in this review, artificial synapses have also been extensively investigated in the applications of neuromorphic electronics and flexible artificial perceptions, which are highly desirable in the development of artificial intelligence, although some issues remain unsolved as shown in Figure 14. For example, the wafer-scale fabrication of artificial synapses with high uniformity, high density, and reliability, using existing processing technology based on low-dimensional materials, is still a challenge, especially on flexible substrates. In addition, artificial synapses with the ability to mimic complicated neuromorphic circuits for complex learning closer to that of biological neural networks was seldom reported. Furthermore, unconventional applications, such as neuromorphic sensory systems, bioelectronic medicines, neuroprosthetics, and soft robotics, need to be further explored to construct intelligent artificial systems.

## Figures and Tables

**Table 1:** Summary of the advantages and disadvantages of low-dimensional materials in the applications of flexible artificial synapses.

	0D Material	1D Material	2D Material
Synthesis of the materials	Easy	Medium	Difficult
Material stability	Medium	High	Low
Type of synaptic device	Memristor	Memristor/Transistor	Memristor/Transistor
Device fabrication	Easy	Difficult	Medium
Wafer scale fabrication	Easy	Difficult	Difficult
Uniformity	Low	Low	High *

* High uniformity in the devices can be realized with wafer-scaled 2D materials.

## Data Availability

Data sharing not applicable.
